# The Relationship Between the Monocyte-to-High-Density Lipoprotein Cholesterol Ratio and Platelet Volume Indices With Diabetic Retinopathy

**DOI:** 10.7759/cureus.79822

**Published:** 2025-02-28

**Authors:** Firdevs Pelin Eskin, Pusem Patir, Ustun Yilmaz, Fulya Duman, Hakan Buber

**Affiliations:** 1 Department of Hematology, University of Health Sciences, Antalya Training and Research Hospital, Antalya, TUR; 2 Department of Nephrology, University of Health Sciences, Antalya Training and Research Hospital, Antalya, TUR; 3 Department of Ophthalmology, University of Health Sciences, Antalya Training and Research Hospital, Antalya, TUR

**Keywords:** diabetes mellitus, diabetic retinopathy, hba1c, monocyte hdl ratio, platelet volume indices

## Abstract

Aim

This study aims to evaluate the roles of monocyte count-to-high-density lipoprotein (HDL) cholesterol ratio (MHR) and platelet volume indices (PVI) in predicting diabetic retinopathy (DR) in patients diagnosed with type 2 diabetes mellitus (DM) and to explore new methods for early prediction of retinopathy.

Methods

This prospective study included 120 patients aged over 18 years with type 2 DM diagnosed according to the American Diabetes Association criteria, along with a control group of 40 healthy individuals. Patients with type 2 DM were divided into three groups: 40 without retinopathy, 40 with non-proliferative diabetic retinopathy (NPDR), and 40 with proliferative diabetic retinopathy (PDR). Parameters such as complete blood count, PVI (mean platelet volume, platelet distribution width, plateletcrit, platelet large cell ratio), lipid profile, inflammatory markers (sedimentation rate, C-reactive protein), MHR, renal function tests, and glucose metabolism markers were analyzed alongside demographic, clinical, and laboratory data to assess their associations with DR in both patient and control groups.

Results

This study investigated the relationship between DR and hematological parameters (PVI, MHR). Hypertension, hyperlipidemia, and chronic kidney disease were common comorbidities. NPDR patients more frequently used metformin and dipeptidyl peptidase (DPP)-4 inhibitors, while PDR patients more often used insulin (p<0.05). Diabetic neuropathy and nephropathy were more prevalent in the PDR group. PDR patients had a significantly longer DM duration. Mean platelet volume (MPV) and platelet large cell ratio (PLCR) were higher in the PDR group. ROC analysis identified MHR (>11.9, 65% sensitivity, 35% specificity) and plateletcrit (PCT<0.29%, 61% sensitivity, 80% specificity) cut-offs for DR prediction. MHR's area under the curve (AUC) was greater than PCT's (p<0.05). MPV differed between PDR and NPDR, but MHR did not differ among DR groups or between DR/non-DR individuals. Glycosylated hemoglobin (HbA1c) and DM duration correlated positively with MHR, which also correlated with leukocyte count. HbA1c and glucose correlated with platelet distribution width (PDW). Each unit increase in DM duration increased MHR 1.13-fold, and each unit increase in HbA1c increased MHR 1.9-fold.

Conclusions

The duration of DM was significantly longer in patients with PDR compared to those with NPDR and those without retinopathy. MPV and PLCR levels were significantly higher in PDR patients than in NPDR patients. In evaluating the relationship between PVI and MHR for predicting diabetic retinopathy, significant cut-off values of MHR and PCT were found to be predictive indicators of diabetic retinopathy.

## Introduction

Diabetes mellitus (DM), recognized as a global health problem by the World Health Organization, is a chronic multisystemic disease implicated in the pathogenesis of damage to multiple tissues and organs. Diabetic retinopathy (DR), one of the most significant complications of DM, is the leading preventable and/or treatable cause of blindness in the 20-65 age group worldwide. Additionally, diabetic nephropathy, an important microangiopathic complication of DM, increases the risk of developing proliferative retinopathy [1]. Establishing a robust classification system is essential for the early diagnosis and determination of treatment indications for DR [2]. DR is classified into two categories: nonproliferative diabetic retinopathy (NPDR) and proliferative diabetic retinopathy (PDR). In NPDR, lesions are confined within the retina, whereas in PDR, lesions extend into the vitreous. This extension can lead to neovascularization in the vitreous, damage to the optic nerve or macula, vitreous hemorrhage, and retinal detachment [3,4].

The risk of severe DR in DM has been associated with the presence of oxidative stress markers [5]. Free radical reactions are a natural consequence of normal metabolic pathways. Oxidant molecules are continuously produced during and after anabolic and catabolic reactions, primarily through glucose oxidation. Simultaneously, antioxidant defense mechanisms have evolved in the body to counteract the harmful effects of free radicals, maintaining a dynamic balance between their formation and neutralization. Monocytes are hematological parameters that increase during inflammation and contribute to oxidative stress [6]. In response to injury, inflammation, or neoplasia and infection, inflammatory monocytes mature into inflammatory macrophages, which participate in an inflammatory response by secreting chemokines and tumor necrosis factor-alpha (TNF-α) [7]. High-density lipoprotein cholesterol (HDL-C) levels decrease in the presence of endothelial dysfunction and atherosclerosis, endowing HDL-C with anti-inflammatory and antioxidant properties [8].

Endothelial dysfunction, increased platelet (PLT) activation, and elevated procoagulant factors are characteristic features of metabolic syndrome. Larger platelets contain denser granules, rendering them metabolically and enzymatically more active than smaller platelets. Consequently, higher thrombotic potential may be associated with increased mean platelet volume (MPV) and platelet distribution width (PDW) [9-12].

In individuals with type 2 DM, new biomarkers are needed to identify and treat those at higher risk of morbidity and mortality from macrovascular and microvascular complications [13]. This study aims to evaluate the roles of monocyte-to-HDL-C ratio (MHR) and platelet volume indices (PVI) in predicting diabetic retinopathy in type 2 DM patients and to investigate new methods for early prediction of retinopathy using simple laboratory parameters.

## Materials and methods

Subjects of the study

This controlled cross-sectional study included 120 patients aged 18 years and older diagnosed with type 2 DM according to the American Diabetes Association criteria, who presented to the internal medicine outpatient clinic of the University of Health Sciences, Antalya Training and Research Hospital between November 2022 and March 2023. Additionally, a control group consisting of 40 healthy individuals was included. The type 2 DM patients were divided into three groups: 40 without retinopathy, 40 with NPDR, and 40 with PDR. In the Early Treatment of Diabetic Retinopathy Study (ETDRS) classification, fundus lesions and characteristics, such as hemorrhages/microaneurysms, venous beading and loops, hard exudates, intraretinal microvascular abnormalities (IRMAs) and neovascularization, were graded individually from standard seven-field 30°C fundus photographs, and based on these individual lesion gradings, an overall retinopathy severity level was determined. Patients with pregnancy, type 1 DM, active/chronic inflammation, active infection, hematological/oncological malignancy, autoimmune diseases, connective tissue diseases, inflammatory bowel disease, acute/chronic kidney disease, acute/chronic liver disease, myeloproliferative diseases, recent blood transfusion, active psychiatric disorders, non-retinopathy eye diseases, those using antiplatelet medications like aspirin or clopidogrel, and those using medications that cause anemia or bone marrow suppression were excluded from the study. The control group was composed of healthy individuals without any health problems.

Clinical assessment and laboratory data

Medical records of the type 2 DM patients included in the study were retrospectively reviewed for gender, age, medical history, anthropometric measurements (height, weight, waist circumference, etc.), physical examination findings, comorbid conditions, duration of DM diagnosis, and medications used. Venous blood samples obtained after an eight-hour overnight fast were evaluated for complete blood count - hemoglobin (Hb), hematocrit (HCT), mean corpuscular volume (MCV), white blood cell count (WBC), neutrophil, monocyte, lymphocyte (PLT), mean platelet volume (MPV), PDW, plateletcrit (PCT), lipid profile (total cholesterol, HDL cholesterol, low-density lipoprotein (LDL) cholesterol, triglycerides), inflammatory markers (sedimentation rate, C-reactive protein), renal function tests (creatinine, urea, uric acid, glomerular filtration rate), and glucose metabolism (fasting glucose, glycosylated hemoglobin, HbA1c). The glomerular filtration rate was estimated using the Modification of Diet in Renal Disease (MDRD) equation. The quantitative urine albumin-to-creatinine ratio was measured in the first-morning urine samples.

Variables and definitions

Plateletcrit is an indicator of platelet mass in circulation, defined as the percentage of platelets in the blood (PLT×MPV/1000). MPV, PDW, platelet large cell ratio (PLCR), and the proportion of platelets larger than 12 femtolitres (fL) to total platelets are used as PVI. The MHR was calculated as an inflammation marker by dividing the monocyte count in 10^3^/µl by HDL cholesterol levels in mmol/L.

Evaluation of retinopathy

All patients were evaluated for diabetic retinopathy by retina specialists using a fundus camera and retinal imaging techniques.

Statistical analysis

It has been calculated that the minimum sample size should be 160, with a power of 0.80 and a type I error rate of 0.05. Data analysis was performed using SPSS version 23.0 (IBM, Inc., Armonk, US). The normality of variables was assessed using visual (histogram and probability plots) and analytical methods (Kolmogorov-Smirnov/Shapiro-Wilk tests). Descriptive analyses for normally distributed variables were presented as mean and standard deviations and intergroup differences were evaluated using analysis of variance (ANOVA). For categorical variables, frequency and percentage values were provided, and differences between groups were assessed using the Chi-square test. The relationship between the biochemical findings of the patients was examined using the Spearman test. Statistical significance was set at p<0.05. Pearson correlation analysis was used to evaluate the relationships between continuous variables. Correlation coefficients (r) and the statistical significance of the relationships (p-value) were calculated. The effect of MHR on DM duration and HbA1c levels was evaluated using regression analysis. The predictive properties of MHR and PVI in forecasting DR were analyzed using receiver operating characteristics (ROC) curve analysis. Sensitivity and specificity values for significant cut-off points were calculated. The area under the curve (AUC) was evaluated, and statistical significance was accepted when the type 1 error rate was below 5%. The odds ratio (OR) with 95% confidence intervals (CI) was calculated.

Ethical statement

This study was approved by the Clinical Research Ethics Committee of the University of Health Sciences, Antalya Training and Research Hospital on 03.11.2022 with decision number 20/10. The study was conducted in accordance with the ethical principles of the Helsinki Declaration.

## Results

Study population

Among the 160 participants in the study, 61 (38%) were male and 99 (62%) were female. The mean age was highest in the NPDR group (64.1±8.07 years) and lowest in the PDR group (55.2±7.9 years), with a significant difference between groups (p<0.05). The duration of DM in the PDR group was 19.6±8.82 years, significantly longer compared to the other groups (p<0.05). More than half of the patients in both the PDR and NPDR groups had hypertension, with hyperlipidemia and chronic kidney disease being the most common comorbidities in these two groups. Regarding medication use, metformin and dipeptidyl peptidase (DPP)-4 inhibitors were more commonly used in the NPDR group, while insulin was more frequently used in the PDR group (p<0.05). The frequency of diabetic neuropathy and nephropathy was significantly higher in the PDR group compared to the other groups. The demographic characteristics, comorbidities, antidiabetic medication histories, lipid profiles, and laboratory findings of all participants are summarized in Table 1.

**Table 1:** Evaluation of demographic characteristics and laboratory findings of individuals * A p-value <0.05 is considered significant. DR - diabetic retinopathy; NPDR - non-proliferative diabetic retinopathy; PDR - proliferative diabetic retinopathy; BMI - body mass index; HT - hypertension; HPL - hyperlipidemia; CKD - chronic kidney disease; SGLT-2 - sodium-glucose cotransporter 2; DPP-4 - dipeptidyl peptidase 4; TZD - thiazolidinedione; ACEI/ARB - angiotensin-converting enzyme inhibitor/angiotensin receptor blocker; β-B - beta blocker; CCB - calcium channel blockers; Hb - hemoglobin; HCT - hematocrit; MCV - mean corpuscular volume; NLR - neutrophil-lymphocyte ratio; MPV - mean platelet volume; PDW - platelet distribution width; PCT - plateletcrit; P-LCR - platelet large cell ratio; MHR - monocyte HDL-C ratio; HDL-C - high-density lipoprotein cholesterol; LDL-C - low-density lipoprotein cholesterol; TC - total cholesterol; FBG - fasting blood glucose; BUN - Blood urea nitrogen; GFR - glomerular filtration rate; CRP - C-reactive protein; DPN - diabetic peripheral neuropathy; DN - diabetic nephropathy

Variables	DR (-) (n=40), mean±SD/n (%)	NPDR (n=40), mean±SD/n (%)	PDR (n=40), mean±SD/n (%)	Control group (n=40), mean±SD/n (%)	p-value*
Age (years)	59.5±12.64^a,b^	64.1±8.07^a^	58.5±10.01^b^	28.9 ±7.16^c^	<0.001
BMI (kg/m^2^)	27.1±5.71^a,b^	26.7±3.65^a^	26.8±5.05^b^	22.0±2.42^c^	<0.001
Duration of DM (years)	10.6±8.39^a^	14.9±6.98^b^	19.6 ±8.82^c^	-	<0.001
Gender					
Female	26 (26.3)^a,b^	22 (22.2)^b^	20 (20.2)^b^	31 (31.3)^a^	0.058
Male	14 (23.0)^a,b^	18 (29.4)^b^	20 (32.8)^b^	9 (14.8)^a^	
Smoking	14 (35.0)	15 (37.5)	14 (35.0)	-	0.316
Comorbidities					
HT	20 (50.0)	27(67.5)	28 (70.0)	-	0.13
HPL	16 (40.0)	12 (30.0)	19 (47.5)	-	0.27
CKD	6 (15.0)	8 (20.0)	12 (30.0)	-	0.25
Medications					
Metformin	26 (65.9)^a,b ^	30 (75.0)^b^	20 (50.0)^a^	-	0
Sulfonylureas	10 (25.0)	10 (25.0)	5 (12.5)	-	0.28
SGLT-2 inhibitor	9 (22.5)	16 (40.0)	14 (35.0)	-	0.22
DPP-4 inhibitor	14(35.0)^a^	23 (57.5)^b^	13 (32.5)^a^	-	0.04
TZD	4 (10.0)	3 (7.5)	5 (12.5)	-	0.75
Insulin	19 (47.5)^a^	24 (60.0)^a,b^	32 (80.0)^b^	-	0.01
ACEI/ARB	15 (37.5)	21 (52.5)	20 (50.0)	-	0.35
β-B	10 (25.0)	10 (25.0)	17 (42.5)	-	0.14
CCB	4 (10.0)	10 (25.0)	9 (22.5)	-	0.18
Diuretic	8 (20.0)	9 (22.5)	15 (37.5)	-	0.16
Statin	14 (35.0)	10 (25.0)	15 (37.5)	-	0.45
Fenofibrate	4 (10.0)	2 (5.0)	5 (12.5)	-	0.49
Laboratory tests					
Hb (g/dL)	12.9±1.38	12.8±1.64	12.4±2.13	13.4±1.27	0.07
HCT (%)	39.4±4.39	38.4±4.66	38.2±6.27	39.9±2.61	0.32
MCV (fL)	84.7±6.82	83.6±5.37	83.8±8.46	86.0±5.37	0.37
WBC (×10^3^/µl)	8.1±1.7	7.8±1.7	8.2±2.1	7.2±1.6	0.08
Neutrophil (×10^3^/µl)	4.9±1.5	4.7±1.3	4.9±1.4	4.2±1.4	0.08
Lymphocyte (×10^3^/µl)	2.3±0.6	2.2±0.7	2.4±1	2.2±0.5	0.7
NLR	2.2±1.03	2.2±0.93	2.3±0.89	1.9±0.75	0.19
Monocyte (×10^3^/µl)	0.60±0.1	0.61±0.1	0.66±0.2	0.54±0.1	0.01
Thrombocyte (×10^3^/µl)	278.2±85.4	266.8±79	269.3±84.6	282.6±59	0.77
MPV (fL)	10.7±0.92^a^	10.2±1.00^b^	10.7±0.94^a^	10.4±0.81^a,b^	0.03
PCT (%)	0.3±0.08	0.2±0.09	0.2±0.11	0.3±0.06	0.12
PDW (fL)	13.2±2.23	12.8 ±2.36	13.2±2.50	12.3±2.09	0.2
P-LCR (%)	32.2±7.23^a^	28.7±7.37^b^	32.8±7.24^a^	29.8±6.56^a,b^	0.03
HDL-C (mmol/L)	51.8±15.09^a,b^	48.9±9.01^a^	48.1±12.79^a^	56.4±10.04^b^	0.009
MHR	12.8±6.00^a^	13.0±3.99^a,b^	15.0±5.65^b^	9.8±2.74^c^	0.00
LDL-C (mmol/L)	113.5±32.01	114.7±25.35	113.8±39.47	109.6±25.69	0.89
TC (mmol/L)	197.7±38.71	196.9±31.02	196.1±53.84	185.8±34.88	0.52
Triglyceride (mmol/L)	173.3±124.0^1a^	164.7±69.36^a^	158.6±105.78^a^	93.8±45.70^b^	0.00
FBG (mg/dl)	180.6±87.39^a^	163.3±67.78^a^	201.8±75.89^a^	82.1±8.67^b^	0.00
HbA1c (%)	9.1±2.74^a^	8.6±1.95^a^	9.4±2.06^a^	5.2±0.23^b^	0.00
BUN (mmol/L)	18.4±10.05^a^	20.3±7.25^a,b^	23.5±11.73^b^	11.8±2.75^c^	0.00
Creatinine (mmol/L)	0.9±0.42^a,c^	1.1±0.59^a,b^	1.2±0.68^b^	0.8±0.14^c^	0.003
GFR (mL/min)	78.8±24.83^a^	69.3±18.51^b^	69.0±24.69^b^	105.3±14.09^c^	0.00
Uric acid (mg/dL)	4.7±1.36^a^	5.0±1.24^a^	5.6±1.21^b^	3.8±1.05^c^	0.00
Sedimentation	14.8±7.69	13.4±6.65	16.3±8.49	12.4±5.94	0.08
CRP (mg/L)	3.3±2.64^a^	2.3±1.32^b,c^	2.7±2.19^a,b^	1.7±1.38^c^	0.003
DPN	12 (30.0)^a^	20 (50.0)^a^	30 (75.0)^b^	-	0
DN	17 (42.5)	17 (42.5)	28 (70.0)^b^	-	0.01

Relationship between retinopathy and PVI, MHR

**Figure 1:**
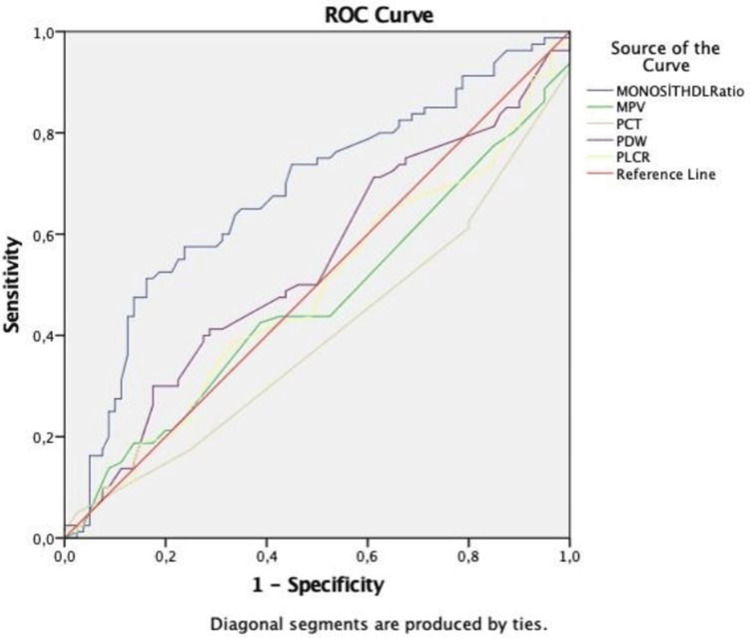
Evaluation of the relationship between the monocyte-to-HDL-C ratio and platelet volume indices in predicting diabetic retinopathy ROC curve analysis was used. MHR - monocyte HDL-C ratio; HDL-C - high-density lipoprotein cholesterol; MPV - mean platelet volume; PDW - platelet distribution width; PCT - plateletcrit; P-LCR - platelet large cell ratio; ROC - receiver operating characteristics

**Figure 2:**
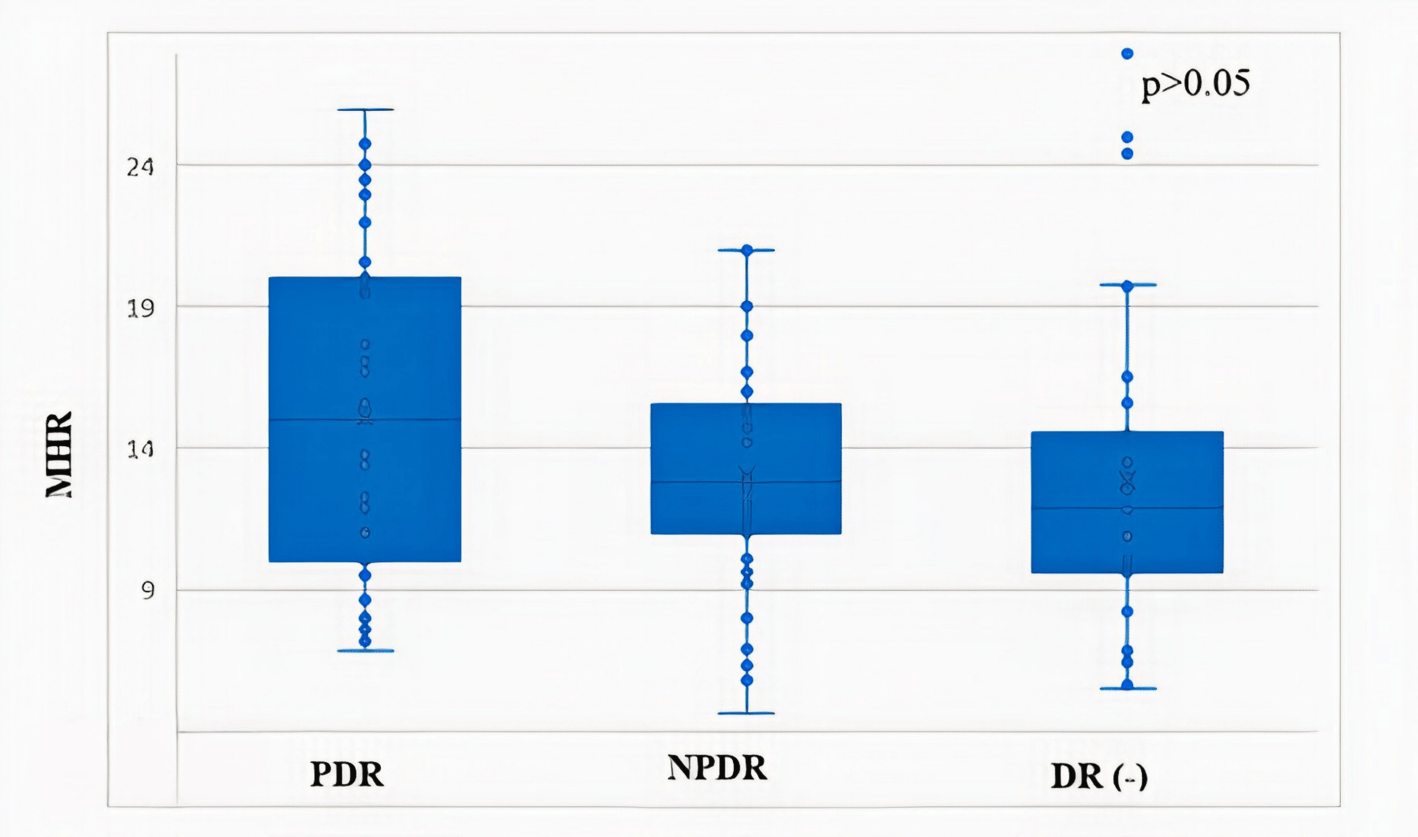
Relationship between the degree of retinopathy and monocyte-to-HDL-C ratio in diabetic patients ANOVA was used for group comparisons. MHR - monocyte HDL-C ratio; HDL-C - high-density lipoprotein cholesterol; DR - diabetic retinopathy; NPDR - non-proliferative diabetic retinopathy; PDR - proliferative diabetic retinopathy; ANOVA - analysis of variance

**Figure 3:**
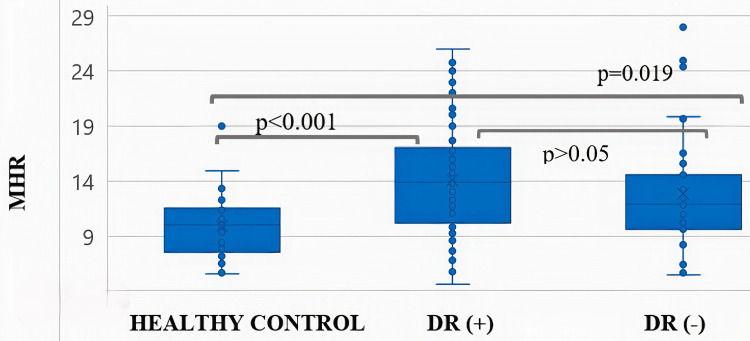
Relationship of monocyte-to-HDL-C ratio in healthy controls, patients with diabetic retinopathy, and patients without diabetic retinopathy ANOVA was used for group comparisons. MHR - monocyte HDL-C ratio; HDL-C - high-density lipoprotein cholesterol; DR - diabetic retinopathy; ANOVA - analysis of variance

PDR exhibited a significantly longer duration of DM compared to those with NPDR or no retinopathy. MPV and PLCR were significantly elevated in the PDR group compared to the NPDR group (Table 1). Receiver operating characteristic (ROC) curve analysis identified statistically significant cut-off values for both MHR and PCT in predicting DR. Specifically, MHR at a cut-off of >11.9 demonstrated a diagnostic sensitivity of 65% and a specificity of 35%, while PCT at a cut-off of <0.29% showed a sensitivity of 61% and a specificity of 80%. The area under the curve for MHR was significantly higher than that for PCT (p<0.05) (Table 2, Figure 1). While MPV was significantly different between PDR and NPDR, no significant difference in MHR values was observed among the PDR, NPDR, and non-retinopathy groups (Figure 2). Furthermore, MHR did not significantly differ between individuals with and without DR (p>0.05; Figure 3).

**Table 2:** Monocyte-to-HDL-C ratio and platelet indices cut-off values AUC - area under curve; MHR - monocyte HDL-C ratio; HDL-C - high-density lipoprotein cholesterol; MPV - mean platelet volume; PDW - platelet distribution width; PCT - plateletcrit; P-LCR - platelet large cell ratio

Parameters	AUC (95% CI)	Cut-off	p-value	Sensitivity (%)	Specificity (%)
MHR	0.680 (0.596-0.764)	11.9	0.000	65.0	35.0
MPV	0.466 (0.376-0.556)	10.1	0.463	44.0	53.0
PDW	0.539 (0.449-0.628)	12.1	0.399	50.0	50.0
PCT	0.398 (0.310-0.485)	0.29	0.025	61.0	80.0
P-LCR	0.485 (0.394-0.575)	29.7	0.735	53.0	53.0

Relationship between HbA1c, DM duration and PVI, MHR

A positive correlation was found between patients' HbA1c and DM duration with MHR. A positive relationship was observed between MHR and leukocyte count. HbA1c and glucose levels were positively correlated with PDW levels (Table 3). With each unit increase in the duration of DM, the ratio of MHR increased by 1.13 times, and with each unit increase in HbA1c levels, MHR increased by 1.9 times (Table 4).

**Table 3:** Correlation between monocyte-to-HDL-C ratio, platelet indices and FBG, HbA1c and duration of diabetes Pearson correlation analysis was used to evaluate the relationships between continuous variables. Correlation coefficients (r) and the statistical significance of the relationships (p) were calculated (*p<0.05, **p<0.01). MPV - mean platelet volume; PDW - platelet distribution width; PCT - plateletcrit; P-LCR - platelet large cell ratio; MHR - monocyte HDL-C ratio; HDL-C - high-density lipoprotein cholesterol; HbA1c - glycated hemoglobin; FBG - fasting blood glucose; DM - diabetes mellitus

		PCT	HGB	WBC	MPV	PDW	P-LCR	MHR	HbA1c	FBG	Duration of DM
PCT	r	1	-0.18^*^	0.20^*^	-0.014	-0.12	-0.07	0	-0.11	-0.004	-0.03
	p		0.023	0.011	0.86	0.11	0.38	0.98	0.13	0.96	0.69
HGB	r		1	0.052	-0.09	-0.058	-0.07	0.04	0.004	-0.03	-0.20^**^
	p			0.51	0.23	0.46	0.35	0.57	0.96	0.64	0.009
WBC	r			1	-0.05	-0.1	-0.07	0.36**	0.07	0.06	0.05
	p				0.52	0.2	0.32	0	0.34	0.44	0.46
MPV	r				1	0.69^**^	0.86^**^	-0.05	0.08	0.06	0.14
	p					0	0	0.51	0.29	0.45	0.06
PDW	r					1	0.84^**^	-0.05	0.17^*^	0.18^*^	0.11
	p						0	0.45	0.02	0.02	0.14
P-LCR	r						1	-0.007	0.13	0.14	0.13
	p							0.92	0.1	0.07	0.08
MHR	r							1	0.28^**^	0.14	0.27^**^
	p								0	0.07	0.001
HbA1c	r								1	0.78^**^	0.42^**^
	p									0	0
FBG	r									1	0.36^**^
	p										0

**Table 4:** Evaluation of the relationship between monocyte-to-HDL-C ratio and diabetes duration and HbA1c levels in diabetic patients Univariate logistic regression analysis assessing the association of MHR with diabetic retinopathy. Stratified by HbA1c levels (<7 vs ≥7%) and diabetes duration (<10 vs ≥10 years). HbA1c - glycated hemoglobin; MHR - monocyte HDL-C ratio; HDL-C - high-density lipoprotein cholesterol

Variables	OR	p-value	95% CI
(Constant)	7.89	0	4.99-10.79
HbA1c			
<7	1		
≥7	1.93	0.04	0.093-3.77
Diabetes duration (years)			
<10	1		
≥10	1.13	0.21	-0.66-2.93

## Discussion

DR is a microvascular complication of DM that can lead to vision loss and blindness. The risk of blindness is significantly elevated in individuals with DM compared to the general population. DR arises from the damage to retinal capillaries, venules, and arterioles due to hyperglycemia or insulin deficiency [1,4].

Monocytes, a hematological parameter, increase during inflammation and contribute to oxidative stress [6]. HDL-C, a lipid parameter, decreases in the presence of endothelial dysfunction and atherosclerosis, exhibiting both anti-inflammatory and antioxidant properties [8]. HDL-C reduces endothelial adhesion molecule expression and exerts antioxidant, anti-inflammatory, and antiplatelet effects through various mechanisms. It also inhibits monocyte activity and macrophage transformation, thus mitigating inflammation. The MHR, derived from these two measurements, is considered a marker of inflammation and oxidative stress [8] and has been investigated in relation to both macrovascular and microvascular diabetic complications.

Studies have reported associations between MHR and cardiovascular disease. Kundi et al. found a correlation between MHR and SYNTAX scores in patients with coronary artery disease [13]. Cetin et al. suggested MHR as an independent marker of coronary artery disease severity and future cardiovascular events in acute coronary syndrome [14]. MHR has also been explored in the context of other diabetic complications. Karatas et al. proposed elevated MHR as a potential biomarker for diabetic nephropathy [15], and Vural et al. reported increased MHR in diabetic axonal polyneuropathy, suggesting its possible use in diagnosing diabetic neuropathy [16]. However, Pençe et al. did not find MHR to be a cardiovascular risk marker specifically in diabetic neuropathy patients, though they observed a correlation with cardiovascular risk across all patients [17].

Regarding DR, Solmaz et al. found a statistically significant difference in MHR between healthy controls and patients with PDR but not between controls and those with NPDR [18]. Tang et al. suggested that MHR in patients with DR was higher than that in both non-DR diabetic patients and healthy controls, while no significant difference was observed in MHR among different DR severity grades [19]. Our study investigated the relationship between MHR and DR stages. We found no significant difference in MHR among PDR, NPDR, and non-retinopathy diabetic patients. While healthy individuals had significantly lower MHR than both DR and non-DR individuals, no significant difference was observed between the MHR of DR and non-DR individuals. MHR demonstrated limited diagnostic utility for distinguishing diabetic retinopathy, potentially reflecting its association with the presence of DM rather than the proliferative process itself. Therefore, our findings indicate that MHR is not a useful marker for PDR.

DM is a prothrombotic condition characterized by endothelial and pericyte damage due to chronic hyperglycemia and insulin resistance, alongside alterations in platelet morphology and function. Platelet hyperactivity contributes to this prothrombotic state [9]. Platelet indices, such as MPV, PDW, and PCT, obtained from simple blood tests, may serve as biomarkers for early detection and prediction of diabetic angiopathies. Platelets play a role in the pathophysiology and progression of atherosclerosis in DM [10-12]. MPV reflects platelet size and is considered a marker of platelet reactivity. Larger platelets are more reactive and prone to activation and aggregation [10-12]. Platelet activation involves a shape change from discoid to spherical, affecting PDW, which measures platelet size variability. A higher PDW suggests increased production of larger, reticulated platelets [10,12,20].

Khanna et al. have shown that the mean MPV, PDW, and P-LCR levels are higher in patients with diabetic retinopathy compared to those without [21]. This finding is consistent with the results of previous studies reporting similar conclusions [22,23]. This study investigated the role of platelet indices in the pathogenesis of DR by evaluating their levels across different DR stages. Consistent with prior research [24,25], we observed significantly elevated MPV and PLCR levels in patients with PDR compared to those with NPDR. Although the precise mechanism underlying elevated MPV in DM remains incompletely elucidated, factors such as hyperglycemia, glucose metabolites, and insulin have been implicated. Potential contributing mechanisms include hyperglycemia-induced osmotic platelet swelling, insulin-stimulated megakaryocyte production of larger platelets, and the presence of larger, younger platelets due to a shortened platelet lifespan. These findings are supported by a meta-analysis conducted by Zaccardi et al. [26], which reported higher MPV and PDW in individuals with DM compared to healthy controls. Our data further suggest a potential role for PLCR in PDR pathogenesis. PLCR, in conjunction with MPV, reflects platelet heterogeneity and has been associated with thrombotic risk. However, the relationship between PCT and DR requires further investigation, as prior studies specifically addressing this association are limited.

This study has several limitations. The small sample size may have limited the power to detect smaller, yet potentially clinically significant, differences. Furthermore, the single-center design may restrict the generalizability of the findings. Additionally, the patient groups were not homogenous with respect to age, BMI, and disease duration, and the use of medications that could affect inflammatory markers represents another limitation.

Future research employing larger, multi-center, prospective designs is recommended to validate these findings and further elucidate the complex relationship between MHR, PVI, and the development and progression of diabetic retinopathy.

## Conclusions

This study demonstrates elevated MPV and PLCR in patients with PDR. Platelet indices may effectively assess platelet dysfunction, potentially providing a predictive measure for retinopathy and serving as a cost-effective monitoring tool for diabetic patients. DM duration was significantly longer in PDR patients compared to those with NPDR and those without retinopathy. A positive correlation was observed between MHR, HbA1c levels, and DM duration. Significant MHR and PCT cut-off values were identified for predicting DR. MHR appears to be influenced by the presence of DM rather than the proliferative process itself, with healthy individuals exhibiting significantly lower MHR compared to diabetic patients. Further multi-center prospective cohort studies with larger sample sizes and longitudinal follow-up are warranted to investigate the relationship between PVI and diabetic microvascular complications, particularly DR and its progression.
